# Anti-MAP Triple Therapy Supports Immunomodulatory Therapeutic Response in Crohn’s Disease through Downregulation of NF-κB Activation in the Absence of MAP Detection

**DOI:** 10.3390/biomedicines8110513

**Published:** 2020-11-18

**Authors:** Ahmad Qasem, Erij Elkamel, Saleh A. Naser

**Affiliations:** Division of Molecular Microbiology, Burnett School of Biomedical Sciences, College of Medicine, University of Central Florida, 4110 Libra Drive, Orlando, FL 32816, USA; ahmadqasem@knights.ucf.edu (A.Q.); erijelkamel@knights.ucf.edu (E.E.)

**Keywords:** Crohn’s disease, IBD, inflammation, cytokines, mycobacterium paratuberculosis, MAP, anti-MAP therapy

## Abstract

We previously reported that the triple antibiotic formulation, known as anti-MAP therapy, exhibits unique synergistic antimicrobial activity and should be effective for treatment of Crohn’s disease (CD) associated with *Mycobacterium avium* subspecies *paratuberculosis* (MAP). The absence of MAP detection in some CD cases may be linked to poor diagnostics or lack of association with the disease. To understand the therapeutic response of some CD patients to anti-MAP therapy in absence of MAP detection, we investigated the immunomodulatory potency of anti-MAP therapy and its major ingredients, clarithromycin (CLA) and rifabutin (RIF), in THP-1, Caco-2, and Jurkat T-cells. Anti-MAP formulation at 2.0 μg/mL decreased MAP viability in macrophages by 18-fold over 72 h. Additionally, M1/M2 macrophage polarization ratio was reduced by 6.7-fold, and expression and protein levels of TNF-α and IL-6 were reduced by 2.9-fold, whereas IL-10 increased by 5.0-fold in these cells. Mechanistically, the effect of anti-MAP formulation on NF-κB p65 activation was dose-dependent and decreased to 13.4% at 2.0 μg/mL. Most importantly, anti-MAP therapy also reversed pro-inflammatory response in lipopolysaccharide (LPS)-induced macrophages, which shows that the anti-inflammatory effect of the treatment is not just due to a decrease in MAP viability. To study the anti-cytotoxic effects of anti-MAP therapy in Caco-2 monolayers infected with MAP or treated with dextran sodium sulfate (DSS), we showed a 45% decrease in lactate dehydrogenase (LDH) activity and an 84% increase in glutathione (GSH) activity, which supports anti-apoptotic activity of the drug. In Jurkat T-cells, anti-MAP therapy decreased T-cell proliferation by 4.8-fold following treatment with phytohemagglutinin (PHA) and by 2.9-fold with MAP purified protein derivative (PPD). Overall, the data demonstrate that anti-MAP therapy plays a significant role in modulating and eliciting a protective immune response in macrophages, endothelial cells, and T lymphocytes, even in absence of infection. This may explain the therapeutic response of some CD patients to treatment, even in absence of MAP detection, infection, or total eradication. The study supports anti-MAP therapy as an alternate treatment option in CD patients, especially in absence of reliable MAP diagnostics.

## 1. Introduction

Several antibiotics have demonstrated anti-inflammatory effects that are independent of their antibacterial properties [1]. Specifically, clinical reports have shown that members of macrolide and rifamycin antibiotic classes have been utilized as useful treatments to suppress inflammation in some dermatological conditions that are unrelated to infectious factors [2]. Clarithromycin (CLA) is a semisynthetic macrolide that exhibits its antibacterial effects through inhibiting protein synthesis by reversibly binding to the peptide exit tunnel of large (50S) ribosomal subunits, thus, preventing peptide chain elongation [3]. It was reported that CLA exhibits anti-inflammatory effects by inhibiting nuclear factor kappa light chain enhancer of activated B cells (NF-κB) activation and pro-inflammatory cytokine production such as of TNF-α, IL-1α, IL-1β, and GM-CSF, and also increasing the production of IL-10 in vitro [4,5]. Additionally, CLA has shown some efficacy in alleviating active Crohn’s disease (CD) symptoms when patients were administered with an oral dosage form of CLA [6].

Rifamycin antibiotics are effective at very low concentrations and exhibit their effects by inhibiting the β-subunit of DNA-dependent RNA polymerases [7]. Studies have shown that rifabutin (RIF) can protect against the emergence of CLA resistance, which is why these two antibiotics are commonly used together [8]. Rifamycins have been shown to have strong anti-inflammatory properties through stimulation of pregnane X receptor (PXR) transcriptional activity and inhibition of NF-κB activation [9]. These effects were observed for the rifamycin antibiotics, rifamycin SV, rifampicin, and rifaximin [9], which are all structurally similar to rifabutin (RIF). As a nuclear receptor, PXR induces expression of cytochrome P450 3A4 (CYP3A4) and P-glycoprotein P (PgP) in the gastrointestinal tract, thus leading to intracellular detoxification [9]. Both of these genes were found to be significantly downregulated in CD patients, and the activation of PXR by rifamycin antibiotics would help maintain intestinal barrier function and reduce inflamed intestinal epithelia [9].

Recently, we have shown that the triple antibiotic drug formulation known as RHB-104, which contains 63.3% CLA, 30% RIF, and 6.7% clofazimine (CLO), has synergistic bactericidal effects against *Mycobacterium avium* subspecies *paratuberculosis* (MAP) [10]. Numerous studies have linked MAP infection to the etiology of CD, and environmental and genetic factors play a major role in increasing susceptibility to MAP infection [11,12,13]. RHB-104 was tested against clinical MAP strains isolated from CD patients, and the minimum inhibitory concentration (MIC) ranged from 0.25 to 10 μg/mL [10]. These concentrations were lower than the MIC of the individual antibiotic components, which ranged from 0.5 to 20 μg/mL [10]. Currently, RHB-104 is subjected to investigation in an FDA approved phase III clinical trial to treat patients with moderate to severe CD [14].

Although a large number of studies strongly support the association between MAP infection and CD pathogenesis, MAP was only detected in up to 70% of clinical samples from CD patients [11,13]. In contrast, other studies have reported lower incidence of MAP infection among CD patients in comparison to healthy controls, which might be due to the fact that different methodology was used for MAP detection in human samples [15]. Detection of MAP in cultures of blood, breast milk, and intestinal tissue was reported using RT-PCR and nested PCR techniques [16]. At least one third of CD patients may not have MAP infection or they may have it at levels below the limit of detection provided by available techniques. Collectively, the lack of more reliable diagnostics or the fact that MAP may not be associated with some CD cases may have contributed to the conflicting reports about the role and significance of MAP in CD etiology. It is even more puzzling that in case studies, some CD patients who were not tested for MAP responded well to antibiotics-based treatments [6]. Regardless of MAP’s role in CD and other autoimmune diseases, the bacterium should be eradicated. This can be effectively achieved using antimicrobial-based therapy including antibiotics. To address these intriguing circumstances, we sought to study treatment options that may offer dual benefits by eradicating MAP infection if it is present, and at the same time reducing inflammation through an immunomodulatory mechanism. Ingredients of RHB-104 triple therapy have been reported to induce a positive therapeutic response in CD patients [17]. Understanding the effect of these antibiotics in stimulated macrophages by infection or antigens would help to elucidate the therapeutic response in CD cases. There is no doubt that treatment options that are based on inhibition of TNF-α by monoclonal antibodies may not provide a long-term therapeutic response, and have also been reported to worsen symptoms in patients associated with infection [18].

A common characteristic of CD patients is overexpressed levels of several pro-inflammatory cytokines and chemokines, as well as significantly higher NF-κB activity compared to healthy individuals [19,20]. The T lymphocytes of CD patients are also characterized by rapid cycling, and restoration of apoptosis by RHB-104 may contribute to reducing inflammation and have positive therapeutic effects [21]. Studies have shown that CD patients exhibit higher levels of enterocyte apoptosis in inflamed areas of the intestine [22], and antibiotic treatment may prevent or reduce oxidative stress in these cells. Therefore, the combined bactericidal and anti-inflammatory properties of CLA and RIF as part of the RHB-104 drug formulation should enhance the efficacy of these antibiotics for CD treatment. Aside from the synergistic bactericidal activity of RHB-104, the present study aims to investigate the synergistic anti-inflammatory and immunomodulatory effects of anti-MAP therapy represented by RHB-104, compared to its individual components, both in the presence and absence of MAP infection in THP-1 macrophages, Caco-2 monolayers, and Jurkat T-cells.

## 2. Materials and Methods

### 2.1. Infection and Treatment of Monocyte-Derived Macrophages

The THP-1 cell line (ATCC TIB-202) was cultured in RPMI-1640 medium (ATCC 30-2001) with 10% fetal bovine serum (FBS; Sigma Life Science, St. Louis, MO, USA). The cells were maintained in a humidified 5% CO_2_ incubator at 37 °C and grown to confluency in cell culture flasks. A total of 1.0 mL of cell suspension was transferred to 12-well tissue culture plates with 1 × 10^5^ cells per well. They were then differentiated into monocyte-derived macrophages using 50 ng/mL phorbol 12-myristate 13-acetate (PMA; Sigma Life Science, St. Louis, MO, USA) followed by 24 h of incubation at 37 °C. Next, monocyte-derived macrophages were treated with 5 µg/mL lipopolysaccharide (LPS) or infected with clinical MAP UCF4 (1 × 10^7^ CFU/mL), followed by 24 h of incubation at the same conditions. Representative of anti-MAP therapy (RHB-104), CLA, and RIF, were kindly provided by RedHill Biopharma Inc. RHB-104 capsule formulation was dissolved in a single proprietary solvent to a final concentration of 1 mg/mL. The stock solution of CLA (1 mg/mL) was prepared by dissolving its powder form in 0.5 M sodium acetate, and the stock solution of RIF (1 mg/mL) was prepared by dissolving its powder form in absolute methanol. Cells were then treated in triplicate with different concentrations of RHB-104 (0.5, 1.0, 2.0 μg/mL) in addition to the corresponding component concentrations of CLA (0.32, 0.63, 1.26 μg/mL), and RIF (0.15, 0.30, 0.60 μg/mL). Cells were incubated for an additional 24 h at the same conditions, and then subjected to further testing. For analysis of *iNOS* and *CD206* expression levels, cells were treated with LPS or infected with MAP and simultaneously treated with antibiotics.

### 2.2. Measurement of MAP Viability in Infected Macrophages

After 24 h of infection with MAP, monocyte-derived macrophages were washed twice with PBS to remove extracellular bacteria, and then antibiotics were added, and the cells were incubated for an additional 24 h. Monocyte-derived macrophages were collected at three time points: 24, 48, and 72 h. The cells were lysed, and then the samples were centrifuged at 14,000 rpm for 20 min. Supernatants were collected and the LIVE/DEAD™ *Bac*Light™ Bacterial Viability Kit (Thermo Fisher Scientific, Waltham, MA, USA) was used according to the manufacturer’s protocol as described earlier [16]. Briefly, 100 µL of each bacterial suspension was pipetted into separate wells of a 96-well flat-bottom microplate in triplicate and mixed with 100 µL of stain solution. The plate was then incubated for 15 min at room temperature (RT) in the dark. Fluorescence was measured using a microplate reader at 530 nm to determine the proportion of live bacteria (green), and at 630 nm to determine the proportion of dead bacteria (red). Data was analyzed by generating a standard plot using fluorescence measurements of five different proportions of live to dead MAP including 0:100, 10:90, 50:50, 90:10, and 100:0. These samples were pipetted in triplicate at volumes of 100 µL and mixed with 100 µL of stain solution. A graph was generated, and the equation of the least squares fit of the relationship between percent live bacteria (x) and green/red fluorescence ratio (y) was used to calculate bacterial viability of the MAP-infected cells treated with antibiotics.

### 2.3. Measurement of NF-κB p65 Transcription Factor Activation in Macrophages

Nuclei were extracted from 1.0 mL of each monocyte-derived macrophage sample suspension after 24 h of antibiotic treatment, using the nuclear extraction kit by Abcam (Cambridge, UK) according to the manufacturer’s protocol. Briefly, cells were centrifuged at 1000 rpm for 5 min and then pellets were suspended in 100 μL of 1X pre-extraction buffer. Following 10 min of incubation on ice, the samples were vortexed for 10 sec and then centrifuged at 12,000 rpm for 1 min. The nuclear pellets were then mixed with 20 μL of extraction buffer containing protease inhibitor cocktail (PIC) and dithiothreitol (DTT) at a 1:1000 ratio. Samples were then incubated on ice for 15 min with periodic vortexing (5 s) every 3 min. Finally, suspensions were centrifuged at 14,000 rpm for 10 min at 4 °C and the supernatants containing nuclear fractions were saved at −80 °C until further use. Protein concentrations were quantified using NanoDrop (OD at 280 nm). The NF-κB p65 Transcription Factor Assay Kit by Abcam (Cambridge, UK) was used to determine NF-κB activation, according to the manufacturer’s protocol. Briefly, nuclear extracts were added to wells, which were coated with a dsDNA sequence specific to NF-κB p65, and the plate was incubated overnight at 4 °C. A transcription factor NF-κB p65 positive control provided by the kit was included as well as a negative control, which omitted NF-κB p65. The plate was then washed 5 times using 1X wash buffer to remove unbound reagents. NF-κB p65 primary antibody was added to each well and the plate was incubated for 1 h on an orbital shaker at RT. Following five washes, diluted transcription factor goat anti-rabbit HRP conjugate secondary antibody was added and incubated for an additional 1 h on an orbital shaker at RT. Following five washes, developing reagents were added to the wells and absorbance was read at 450 nm. Percent activation scores were calculated by the equation: (sample absorbance reading/positive control absorbance reading) × 100.

### 2.4. Measurement of iNOS, CD206, TNF-α, IL-6, and IL-10 Expression in Treated Macrophages

RNA was isolated from each 1.0 mL sample of monocyte-derived macrophages following 24 h of antibiotic treatment. RNA was then reverse-transcribed to cDNA, then gene expression was measured using specific primers for *GAPDH*, *iNOS*, *CD206*, *TNF-α*, *IL-6*, and *IL-10* obtained from Invitrogen (Carlsbad, CA, USA) (Table 1), followed by quantitative reverse transcription PCR (RT-qPCR) analysis. To isolate RNA, cells were centrifuged at 2500 rpm for 5 min at 4 °C and then pellets were suspended in 500 µL of TRIzol reagent (Invitrogen, Carlsbad, CA, USA) and left at RT for 5 min. Next, 125 µL of chloroform was added to each sample followed by an additional 5 min incubation period at RT. The samples were then centrifuged at 10,000 rpm for 5 min at 4 °C and the top clear aqueous layers containing RNA were transferred to new 2.0 mL microcentrifuge tubes. These were then gently mixed with 275 µL of 100% isopropanol, incubated at RT for 5 min, and then centrifuged at 14,000 rpm for 20 min at 4 °C. Next, the samples were placed on ice and the isopropanol was poured off, and 500 µL of 75% ethanol in DEPC-treated water was added. The samples were centrifuged at 9500 rpm for 5 min at 4 °C. Ethanol was then poured off and the pellets were left to air-dry for 10 min. Finally, 15 µL of DEPC-treated water was added to each tube and gently mixed. RNA concentrations were measured using NanoDrop (OD at 260 nm). Next, cDNA was synthesized from 800 ng of each RNA sample, 4 µL of iScript reverse transcription supermix (Bio-Rad, Hercules, CA, USA), and then topped up to a total volume of 20 µL with RNase-free water. A thermal cycler (MyGene Series Peltier Thermal Cycler) was used to perform the reactions for 5 min at 25 °C, 20 min at 46 °C, and 1 min at 95 °C. The cDNA samples were either stored at −20 °C or used immediately for RT-qPCR analysis. For each sample, 1 µL of cDNA was mixed with 10 µL of Fast SYBR Green Master Mix (Thermo Fisher Scientific, Waltham, MA), 1 µL of forward primer, 1 µL of reverse primer (see Table 1), and 7 µL of DEPC-treated water. Samples were added in triplicate to a 96-well microamp RT-PCR reaction plate and the experiment was run using 7500 Fast Real-Time PCR System (Applied Biosystems, Foster City, CA, USA). *GAPDH* was used as a control to get baseline C_T_ values, and gene expression of *iNOS*, *CD206*, *TNF-α*, *IL-6*, and *IL-10* were measured. Relative gene expression levels were calculated using: $(2^{-\Delta \mathrm{CT}})\times 1000$, where $\Delta \mathrm{CT}={\mathrm{CT}}_{\mathrm{sample}}\;-{\;\mathrm{CT}}_{\mathrm{GAPDH}}$.

### 2.5. Measurement of TNF-α, IL-6, and Il-10 Protein Expression in Treated Macrophages

Following 24 h of antibiotic treatment, monocyte-derived macrophages were pelleted by centrifugation at 2500 rpm for 5 min at 4 °C. The supernatants were saved and TNF-α, IL-6, and Il-10 protein levels were determined using the following ELISA kits: Human TNF-α High Sensitivity ELISA, Human IL-6 High Sensitivity ELISA, and Human IL-10 High Sensitivity ELISA, all of which were purchased from Invitrogen (Carlsbad, CA, USA). All groups were tested in triplicate.

### 2.6. Caco-2 Cell Culture and Treatment

The antioxidant effects of antibiotics were evaluated in a human enterocyte-like cell line (Caco-2 ATCC HTB-37). Cells were routinely cultured in ATCC-formulated Eagle’s Minimum Essential Medium (EMEM) supplemented with 20% FBS (ATCC, Manassas, VA, USA) and maintained at 37 °C in a humidified 5% CO_2_ incubator. Cells were grown in 12-well plates at a density of 3 × 10^5^ cells per well until confluency and differentiation were reached in 21 days. Fully differentiated Caco-2 monolayers in 12-well plates were infected with 1 × 10^7^ CFU/mL of MAP UCF4 or 3% dextran sodium sulfate (DSS) for 24 h in the presence of either RHB-104 (1.0 μg/mL), CLA (0.63 μg/mL), or RIF (0.30 μg/mL). Caco-2 monolayers were then lysed, and lactate dehydrogenase (LDH) and glutathione (GSH) levels were measured.

### 2.7. Trypan Blue Exclusion Assay in Caco-2 Monolayers

We evaluated the ability of RHB-104 and its major components to prevent cytotoxic effects caused by MAP in Caco-2 monolayers using a trypan blue exclusion assay (Thermo Fisher, Waltham, MA, USA). Following 24 h of infection and antibiotic treatment, supernatant was removed, and monolayers were washed with fresh media. Next, 10 μL of trypan blue solution was added to each sample and monolayers were incubated at RT for 30 min. Finally, trypan blue was removed, monolayers were examined under the microscope, and pictures were taken at 100 × magnification.

### 2.8. Measuring LDH Activity in Caco-2 Monolayers

We used the CyQUANT LDH cytotoxicity assay (Invitrogen, Carlsbad, CA, USA), a fluorescent-based method to quantify cellular LDH cytotoxicity levels. Briefly, following the manufacturer instructions, two sets of triplicate wells in a 12-well plate were prepared. Following MAP infection or DSS treatment in the presence of antibiotics for 24 h, one set of each treated monolayers was lysed and used to determine maximum LDH activity, and the second set was used to determine spontaneous LDH activity. A total of 50 μL of each sample medium and positive control was transferred into a 96-well plate in triplicate and then mixed with 50 μL of reaction mixture. After a 10 min incubation at RT, 50 μL of stop solution was added and fluorescence was measured using an excitation of 560 nm and an emission of 590 nm. Cytotoxicity was calculated using the formula: (treated sample LDH − spontaneous LDH)/(maximum LDH − spontaneous LDH) × 100. Each value was divided by the positive control value to determine the percent decrease in LDH activity.

### 2.9. Measuring Reduced GSH Levels in Caco-2 Monolayers

To determine GSH levels following MAP infection or DSS treatment in the presence of antibiotics, we used the reduced GSH assay kit (Sigma Life Science, St. Louis, MO, USA), according to the manufacturer’s instructions. Briefly, 100 μL of collected cell pellets were mixed with 100 μL of 5% sulfosalicylic acid solution and vortexed vigorously. Samples were then centrifuged at 12,000× *g* at 4 °C for 20 min. Supernatants were collected, followed by a 5-fold dilution in GSH assay buffer, and then 10 μL were transferred into a 96-well plate in triplicate. Six serial dilutions were prepared from the GSH positive control provided by the kit and added to the plate in order to generate a standard curve. Finally, 80 μL of reaction mix were added to each well and absorbance was measured at 450 nm. The amount of GSH in nmol/mg was calculated from the generated curve equation.

### 2.10. DHE Fluorescence Staining Assay

DHE fluorescence staining was performed on Caco-2 monolayers following MAP infection and antibiotics treatment for 24 h. Monolayers were washed twice with cold PBS, and then fixed with 4% paraformaldehyde (PFA) for 15 min. Monolayers were then washed twice with cold PBS, and then treated with 1 μM DHE stain (Sigma Aldrich, St. Louis, MO, USA) for 25 min. Next, 60 μL VECTASHIELD Antifade Mounting Medium containing 4′,6-diamidino-2-phenylindole (DAPI; Vector Laboratories, Burlingame, CA, USA) was used to co-stain nuclei. Slides were examined under AmScope IN480TC-FL-MF603 Fluorescence Microscope, where red staining indicates oxidative stress and blue staining represents nuclei. Captured images were analyzed by measuring average integrated density using NIH Image J 1.39o software (Rockville, MD, USA), which was also used to generate merged images.

### 2.11. T-Cell Proliferation Assay Following Antibiotic Treatment

The Jurkat T-cell line (ATCC TIB-152) was used and cultured in RPMI-1640 medium (ATCC 30-2001) with 10% FBS (Sigma Life Science, St. Louis, MO, USA). The cells were maintained in a humidified 5% CO_2_ incubator at 37 °C and grown to confluency in cell culture flasks. Moreover, 2 × 10^5^/mL of isolated T-cells were transferred to a 96-well culture plate with 100 μL aliquots per well and then incubated either with RPMI only, 10 μg/mL phytohemagglutinin (PHA; Sigma Life Science, St. Louis, MO, USA), or 5 μg/mL MAP purified protein derivative (PPD), and concurrently treated in triplicate with 1.0 μg/mL RHB-104, 0.63 μg/mL CLA, or 0.30 μg/mL RIF. MAP PPD was prepared from sonicates of protein extracts from cell pellets of MAP UCF4, followed by centrifugation at 16,000 rpm for 30 min, and then isolation of the supernatant containing PPD. The cells were incubated for 48 h in a humidified 5% CO_2_ incubator at 37 °C. A T-cell proliferation assay was performed using the bromodeoxyuridine (BrdU) labeling proliferation ELISA kit by Abcam (Cambridge, UK) as per the manufacturer’s protocol. Briefly, T-cells were labelled using 20 μL of 1X BrdU and incubated for an additional 24 h at 37 °C under the same conditions. Then, 200 μL of fixing solution was added to each well and then incubated for 30 min at RT. The plate was then washed 3 times using 1X wash buffer. Next, 100 μL of anti-BrdU monoclonal detector antibody was added to each well and the plate was incubated for 1 hour at RT. The wells were washed and then 100 μL of 1X peroxidase goat anti-mouse IgG conjugate was added to each well, and the plate was incubated at RT for 30 min. Following another wash step, a total of 100 μL of TMB peroxidase substrate was added to each well and the plate was incubated in the dark for 30 min at RT. Lastly, 100 μL of stop solution was added to the wells and absorbance was read at 450 nm using a spectrophotometric microtiter plate. Relative T-cell proliferation of samples compared to controls (T-cells with RPMI only) were determined by calculating fold change in absorbance readings at 450 nm.

## 3. Statistical Analysis

GraphPad Prism V.7.02 (GraphPad, La Jolla, CA, USA) was used to analyze data statistics. All data collected in this study were pre-tested for normal distribution using the Kolmogorov–Smirnov normality test. The unpaired two-tailed t-test was used to determine significance. Data are expressed as average ± SD of the mean, and the difference between treated samples vs. controls was considered statistically significant at a level of *p* value < 0.05 and at 95% confidence interval (CI).

## 4. Results

### 4.1. RHB-104 Decreases MAP Survival in Infected Macrophages at Lower Concentrations than CLA and RIF

**Figure 1:**
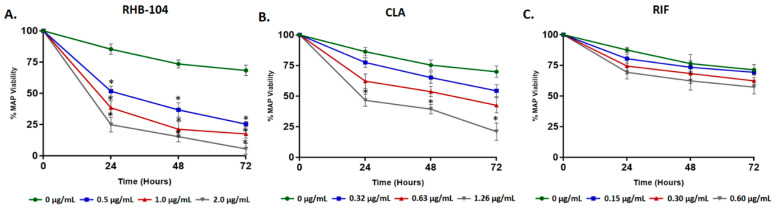
Effect of representative of anti-MAP therapy (RHB-104) and its major components on *Mycobacterium avium* subspecies *paratuberculosis* (MAP) viability in infected macrophages. MAP viability was measured in infected macrophages following treatment with RHB-104 (0, 0.5, 1.0, 2.0 μg/mL) (**A**), clarithromycin (CLA) (0, 0.32, 0.63, and 1.26 μg/mL) (**B**), and rifabutin (RIF) (0, 0.15, 0.30, 0.60 μg/mL) (**C**). * *p* < 0.05.

We tested the effects of RHB-104 (0.5, 1.0, and 2.0 μg/mL) and its individual components, CLA (0.32, 0.63, and 1.26 μg/mL), and RIF (0.15, 0.30, and 0.60 μg/mL) on modulating MAP viability in infected macrophages at three time points over 72 h. Our results indicate that RHB-104 at 0.5, 1.0, and 2.0 μg/mL reduced MAP viability significantly in a concentration-dependent manner over time (Figure 1A). At 2.0 μg/mL RHB-104, MAP viability declined from 100% to 29.97%, 15.37%, and 5.51% after 24, 48, and 72 h of treatment, respectively. The effect of CLA on MAP viability was only significant at 1.26 μg/mL, where MAP viability declined from 100% to 38.49%, 21.26%, and 17.58%, following 24, 48, and 72 h of treatment, respectively (Figure 1B). However, RIF did not affect MAP viability significantly at all three concentrations tested (Figure 1C).

### 4.2. RHB-104 Significantly Reduces M1/M2 Macrophage Polarization at Lower Concentrations than Its Major Components

**Figure 2:**
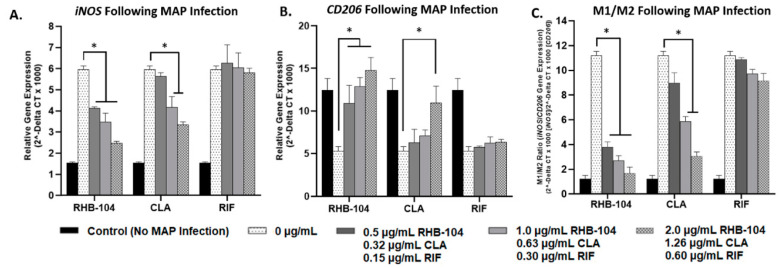
Effect of RHB-104 and its major components on M1/M2 macrophage polarization in MAP-infected macrophages. RT-PCR was used to measure expression of (**A**) *iNOS* (M1), and (**B**) *CD206* (M2) in MAP-infected macrophages (24 h) following dose-dependent treatment with RHB-104 (0, 0.5, 1.0, 2.0 μg/mL), CLA (0, 0.32, 0.63, and 1.26 μg/mL), and RIF (0, 0.15, 0.30, 0.60 μg/mL) for 24 h. (**C**) Illustrates the calculated *iNOS*/*CD206* (M1/M2) ratio for all treatments. * *p* < 0.05.

**Figure 3:**
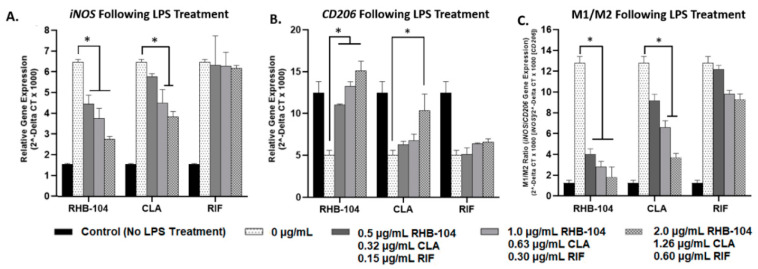
Effect of RHB-104 and its major components on M1/M2 Macrophage Polarization in lipopolysaccharide (LPS)-treated macrophages. RT-PCR was used to measure expression of (**A**) *iNOS* (M1), and (**B**) *CD206* (M2) in LPS-treated macrophages (24 h) following dose-dependent treatment with RHB-104 (0, 0.5, 1.0, 2.0 μg/mL), CLA (0, 0.32, 0.63, and 1.26 μg/mL), and RIF (0, 0.15, 0.30, 0.60 μg/mL) for 24 h. (**C**) illustrates the calculated *iNOS*/*CD206* (M1/M2) ratio for all treatments. * *p* < 0.05.

The effects of RHB-104 (0.5, 1.0, and 2.0 μg/mL), CLA (0.32, 0.63, and 1.26 μg/mL), and RIF (0.15, 0.30, and 0.60 μg/mL) were examined on the expression of the pro-inflammatory M1 macrophage marker, *iNOS*, and the anti-inflammatory M2 macrophage marker, *CD206*, following MAP infection or LPS treatment for 24 h. MAP-infected macrophages with no antibiotic treatment exhibited the highest levels of *iNOS* (5.97 ± 0.17) and the lowest levels of *CD206* (5.33 ± 0.51); these groups were used as reference controls. RHB-104 caused significant reductions in *iNOS* expression at all concentrations tested in MAP-infected macrophages (4.13 ± 0.07, 3.49 ± 0.41, and 2.47 ± 0.95 at 0.5, 1.0, and 2.0 μg/mL, respectively) (Figure 2A). However, CLA only caused significant reductions in *iNOS* expression at 0.63 and 1.26 μg/mL, but not at 0.32 μg/mL (4.18 ± 0.51 and 3.35 ± 0.14 at 0.63 and 1.26 μg/mL, respectively) (Figure 2A). RIF did not cause significant reduction in *iNOS* at any of the concentrations tested in MAP-infected macrophages (Figure 2A). Additionally, RHB-104 caused significant upregulation of *CD206* expression in MAP-infected macrophages at all concentrations tested (10.90 ± 2.11, 12.85 ± 1.07, and 14.8 ± 0.92 at 0.5, 1.0, and 2.0 μg/mL, respectively) (Figure 2B). However, CLA only caused significant upregulation of *CD206* at a concentration of 1.26 μg/mL (10.95 ± 1.96) and RIF did not significantly affect *CD206* expression (Figure 2B). These results are also reflected in the *iNOS*/*CD206* (M1/M2) ratios, which were significantly reduced in response to RHB-104 at all concentrations tested (3.79 ± 0.44, 2.71 ± 0.40, and 1.68 ± 0.50 at 0.5, 1.0, and 2.0 μg/mL, respectively) compared to MAP-infected macrophages with no antibiotic treatment (11.20 ± 0.34). CLA only exhibited significant reduction in M1/M2 following treatment with 0.63 and 1.26 μg/mL (5.86 ± 0.41 and 3.06 ± 0.34, respectively) (Figure 2C). Similar trends were exhibited in response to antibiotic treatment in LPS-treated macrophages. Macrophages treated with LPS and with no antibiotic treatment exhibited the highest levels of *iNOS* (6.45 ± 0.12) and the lowest levels of *CD206* (5.04 ± 0.56); these groups were used as reference controls. RHB-104 caused significant downregulation of *iNOS* expression at all concentrations tested (4.43 ± 0.43, 3.74 ± 0.48, and 2.74 ± 0.14 at 0.5, 1.0, and 2.0 μg/mL, respectively) and significant upregulation in *CD206* at all concentrations tested (11.02 ± 0.13, 13.24 ± 0.57, and 15.08 ± 0.97 at 0.5, 1.0, and 2.0 μg/mL, respectively) in LPS-treated macrophages (Figure 3A,B). CLA only caused significant downregulation of *iNOS* expression at 0.63 and 1.26 μg/mL (4.49 ± 0.64 and 3.83 ± 0.25, respectively), and significant upregulation of *CD206* only at 1.26 μg/mL (10.36 ± 1.96) in LPS-treated macrophages (Figure 3A,B). RIF did not significantly change *iNOS* or *CD206* expression at any of the concentrations tested (Figure 3A,B). Additionally, M1/M2 ratios were significantly reduced in response to RHB-104 treatment at all concentrations tested (4.02 ± 0.53, 2.83 ± 0.51, and 1.82 ± 0.97 at 0.5, 1.0, and 2.0 μg/mL, respectively) compared to LPS-treated macrophages with no antibiotics (12.80 ± 0.62), followed by CLA only at the upper two concentrations tested (6.60 ± 0.64 and 3.70 ± 0.41 at 0.63 and 1.26 μg/mL, respectively (Figure 3C).

### 4.3. RHB-104 Decreases NF-κB p65 Activation in Macrophages More Effectively than Its Major Components

NF-κB p65 activation was determined in MAP-infected and LPS-treated macrophages treated with RHB-104, CLA, and RIF (Table 2). Activation scores represent the proportion of activated NF-κB p65, as indicative by nuclear translocation, in comparison to the positive control. Our results showed that RHB-104 exhibited reduced NF-κB p65 activation in a concentration-dependent manner at 0.5, 1.0, and 2.0 μg/mL in MAP-infected macrophages (48.4%, 21.5%, and 13.4%, respectively) and LPS-treated macrophages (67.2%, 37.8%, and 23.3%, respectively). Clarithromycin also reduced NF-κB p65 activation at 0.32, 0.63, and 1.26 μg/mL in MAP-infected macrophages (58.3%, 49.6%, and 24.1%, respectively) and LPS-treated macrophages (68.2%, 57.2%, and 33.6%, respectively). However, the activation scores in response to CLA treatment exhibited lower levels of reduction than RHB-104 treatment at comparable concentrations. Rifabutin caused reduced NF-κB p65 activation in a concentration-dependent manner as well; however, the effects were not as strong as those seen by RHB-104 and CLA treatment. At 0.15, 0.30, and 0.60 μg/mL, RIF caused activation scores of 83.8%, 73.1%, and 57.5%, respectively, in MAP-infected macrophages, and 89.1%, 83.4%, and 68.7%, respectively, in LPS-treated macrophages.

### 4.4. RHB-104 Is Superior to Clarithromycin and Rifabutin at Modulating Cytokine Gene Expression

**Figure 4:**
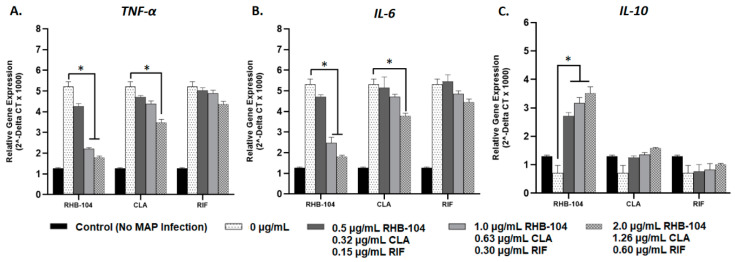
Effect of RHB-104 and its major components on *TNF-α*, *IL-6*, and *IL-10* expression in MAP-infected macrophages. RT-PCR measurements of expression of *TNF-α* (**A**), *IL-6* (**B**), and *IL-10* (**C**) in MAP-infected macrophages (24 h) followed by treatment with RHB-104 (0, 0.5, 1.0, 2.0 μg/mL), CLA (0, 0.32, 0.63, and 1.26 μg/mL), and RIF (0, 0.15, 0.30, 0.60 μg/mL) for 24 h. * *p* < 0.05.

**Figure 5:**
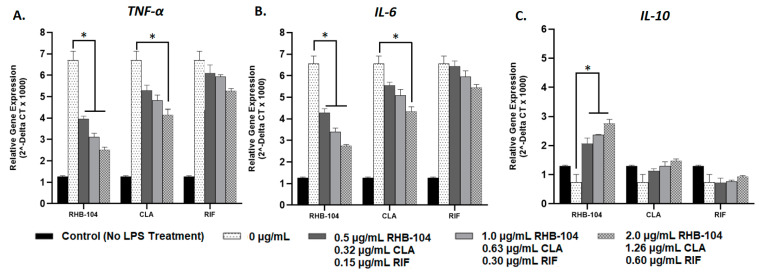
Effect of RHB-104 and its major components on *TNF-α*, *IL-6*, and *IL-10* expression in LPS-treated macrophages. RT-PCR measurements of expression of *TNF-α* (**A**), *IL-6* (**B**), and *IL-10* (**C**) in LPS-treated macrophages (24 h) followed by treatment with RHB-104 (0, 0.5, 1.0, 2.0 μg/mL), CLA (0, 0.32, 0.63, and 1.26 μg/mL), and RIF (0, 0.15, 0.30, 0.60 μg/mL) for 24 h. * *p* < 0.05.

Relative gene expression of pro-inflammatory cytokines, *TNF-α* and *IL-6*, and the anti-inflammatory cytokine, *IL-10*, were measured using RT-PCR in response to RHB-104 (0.5, 1.0, and 2.0 μg/mL), CLA (0.32, 0.63, and 1.26 μg/mL), and RIF (0.15, 0.30, and 0.60 μg/mL) antibiotic treatment in MAP-infected and LPS-treated macrophages. Expression of *TNFα* and *IL-6* were highest (5.20 ± 0.25 and 5.30 ± 0.27, respectively), and *IL-10* expression was lowest (0.71 ± 0.27) in MAP-infected macrophages with no antibiotic treatment; these groups were used as reference controls (Figure 4). Pro-inflammatory cytokine gene expression was significantly reduced in response to RHB-104 treatment at concentrations of 1.0 and 2.0 μg/mL in MAP-infected macrophages. Specifically, *TNF-α* gene expression levels were 2.21 ± 0.07 and 1.79 ± 0.07 at 1.0 and 2.0 μg/mL, respectively, and *IL-6* expression levels were 2.47 ± 0.28 and 1.81 ± 0.07 at 1.0 and 2.0 μg/mL, respectively (Figure 4A,B). However, CLA only exhibited significant reduction in *TNF-α* and *IL-6* expression at a concentration of 1.26 μg/mL (3.47 ± 0.16 and 3.78 ± 0.13, respectively) (Figure 4A,B). In contrast, RIF did not exhibit significant changes in gene expression in *TNF-α* or *IL-6* (Figure 4A,B). Furthermore, expression of the anti-inflammatory cytokine, *IL-10*, was significantly increased in response to RHB-104 at all concentrations tested (2.71 ± 0.13, 3.17 ± 0.20, and 3.52 ± 0.22 at 0.5, 1.0, and 2.0 μg/mL, respectively) (Figure 4C). Both CLA and RIF did not exhibit significant changes in *IL-10* expression at any of the tested concentrations (Figure 4C). In parallel to these results, similar findings were observed in LPS-treated macrophages in response to the same antibiotic treatments. Expression of *TNF-α* and *IL-6* were highest (6.70 ± 0.42 and 6.55 ± 0.36, respectively), and *IL-10* expression was lowest (0.74 ± 0.27) in LPS-treated macrophages with no antibiotic treatment; these groups were used as reference controls (Figure 5). As shown in Figure 5A, RHB-104 caused significant reduction in *TNF-α* expression at all concentrations tested (3.96 ± 0.13, 3.12 ± 0.17, and 2.52 ± 0.12 at 0.5, 1.0, and 2.0 μg/mL, respectively). RHB-104 also significantly reduced *IL-6* expression at all concentrations (4.28 ± 0.189, 3.40 ± 0.17, and 2.75 ± 0.06 at 0.5, 1.0, and 2.0 μg/mL, respectively) (Figure 5B). However, CLA only significantly reduced expression of *TNF-α* and *IL-6* at 1.26 μg/mL (4.15 ± 0.27 and 4.35 ± 0.21, respectively) in LPS-treated macrophages (Figure 5A,B). RIF did not cause significant changes in *TNF-α* or *IL-6* expression. *IL-10* expression levels were only significantly reduced by RHB-104 (2.07 ± 0.20, 2.36 ± 0.02, 2.76 ± 0.14 at 0.5, 1.0, and 2.0 μg/mL, respectively), but no significant changes were observed in response to CLA or RIF antibiotic treatments in LPS-treated macrophages (Figure 5C).

### 4.5. RHB-104 Is Superior to Clarithromycin and Rifabutin at Modulating Cytokine Protein Levels

We evaluated cytokine levels in MAP-infected and LPS-treated macrophages in response to treatment with RHB-104 (0.5, 1.0, and 2.0 μg/mL), CLA (0.32, 0.63, and 1.26 μg/mL), and RIF (0.15, 0.30 and 0.60 μg/mL) using an ELISA-based approach (Table 3). TNF-α and IL-6 levels in MAP-infected macrophages with no antibiotic treatment were 191.2 ± 8.6 pg/mL and 201.8 ± 11.7 pg/mL, respectively, and IL-10 levels were 61.9 ± 9.8 pg/mL. Relative to these MAP controls, RHB-104 showed significant downregulation of TNF-α at 1.0 and 2.0 µg/mL with 123.7 ± 6.7 pg/mL and 95.8 ± 1.8 pg/mL, respectively. However, CLA only showed a significant decrease in TNF-α at 1.26 µg/mL (146.1 ± 6.2 pg/mL), and RIF showed no significant change in TNF-α. Similarly, IL-6 levels were significantly reduced in response to 1.0 and 2.0 µg/mL RHB-104 (139.4 ± 3.2 pg/mL and 117.5 ± 2.5 pg/mL, respectively). CLA only significantly reduced IL-6 at 1.26 µg/mL (153.2 ± 4.6 pg/mL), and RIF did not cause significant changes in IL-6 in MAP-infected macrophages. The anti-inflammatory cytokine, IL-10, was significantly increased in response to 1.0 and 2.0 µg/mL RHB-104 (88.6 ± 3.5 pg/mL and 95.1 ± 5.8 pg/mL, respectively), but both CLA and RIF did not significantly change IL-10 levels at any of the concentrations tested. Comparably, similar trends were observed in LPS-treated macrophages. LPS controls with no antibiotic treatment exhibited TNF-α and IL-6 levels of 217.4 ± 5.1 pg/mL and 224.3 ± 9.6 pg/mL, respectively, and IL-10 levels of 52.2 ± 7.4 pg/mL. These control groups were used as reference standards for the groups with antibiotic treatment. RHB-104 caused a relatively significant decrease in TNF-α at 1.0 and 2.0 µg/mL (139.7 ± 4.7 pg/mL and 110.9 ± 4.0 pg/mL, respectively) and in IL-6 at 1.0 and 2.0 µg/mL (151.1 ± 2.5 pg/mL and 137.2 ± 2.7 pg/mL, respectively). However, CLA only caused significant reductions in TNF-α and IL-6 at 1.26 µg/mL (158.2 ± 7.3 pg/mL and 169.7 ± 1.1 pg/mL, respectively). RIF did not significantly change TNF-α or IL-6 levels at any of the concentrations tested. Lastly, IL-10 was significantly increased in response to 1.0 and 2.0 µg/mL RHB-104 (84.3 ± 6.9 pg/mL and 91.91 ± 5.2 pg/mL, respectively), but IL-10 did not significantly change in response to CLA or RIF treatment at any of the tested concentrations.

### 4.6. RHB-104 Synergistically Reduces Tissue Damage and Oxidative Stress in Caco-2 Monolayers

**Figure 6:**
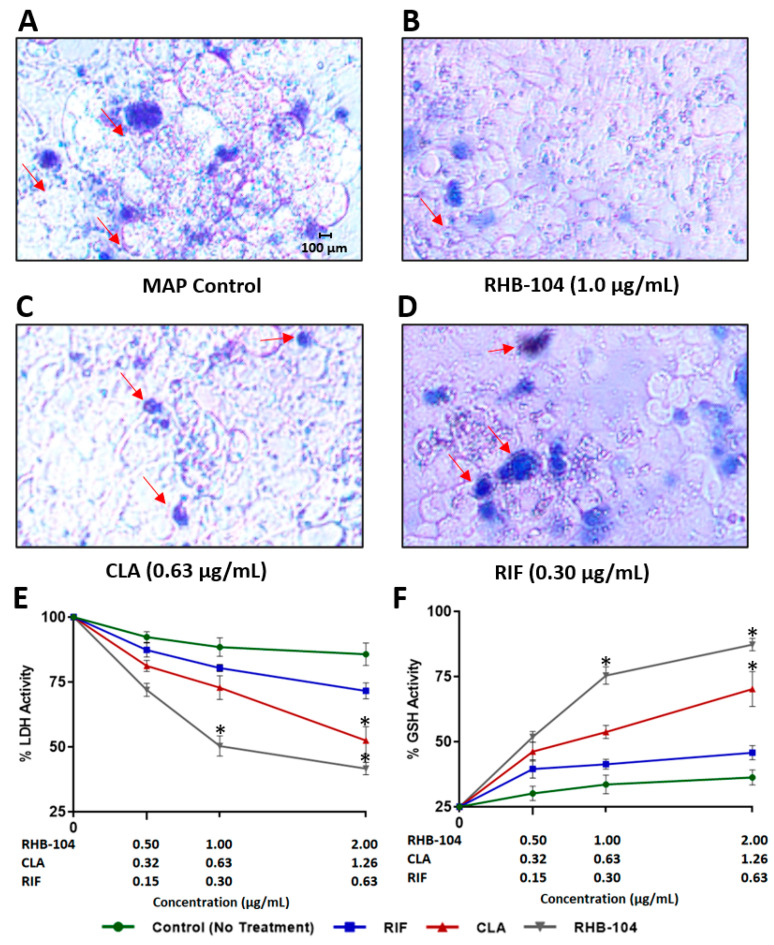
Anti-cytotoxic activity of RHB-104 and its major components in Caco-2 monolayers infected with MAP. Trypan blue exclusion assay showing the cytotoxic effects (indicated by red arrows) in MAP-infected Caco-2 monolayers (**A**), and MAP-infected Caco-2 monolayers treated with 1.0 μg/mL RHB-104 (**B**), 0.63 μg/mL CLA (**C**), and 0.30 μg/mL RIF (**D**). LDH Activity (**E**) and GSH Activity (**F**) in MAP-infected Caco-2 monolayers treated with RHB-104 (0, 0.5, 1.0, 2.0 μg/mL), CLA (0, 0.32, 0.63, and 1.26 μg/mL), and RIF (0, 0.15, 0.30, 0.60 μg/mL) for 24 h. * *p* < 0.05.

**Figure 7:**
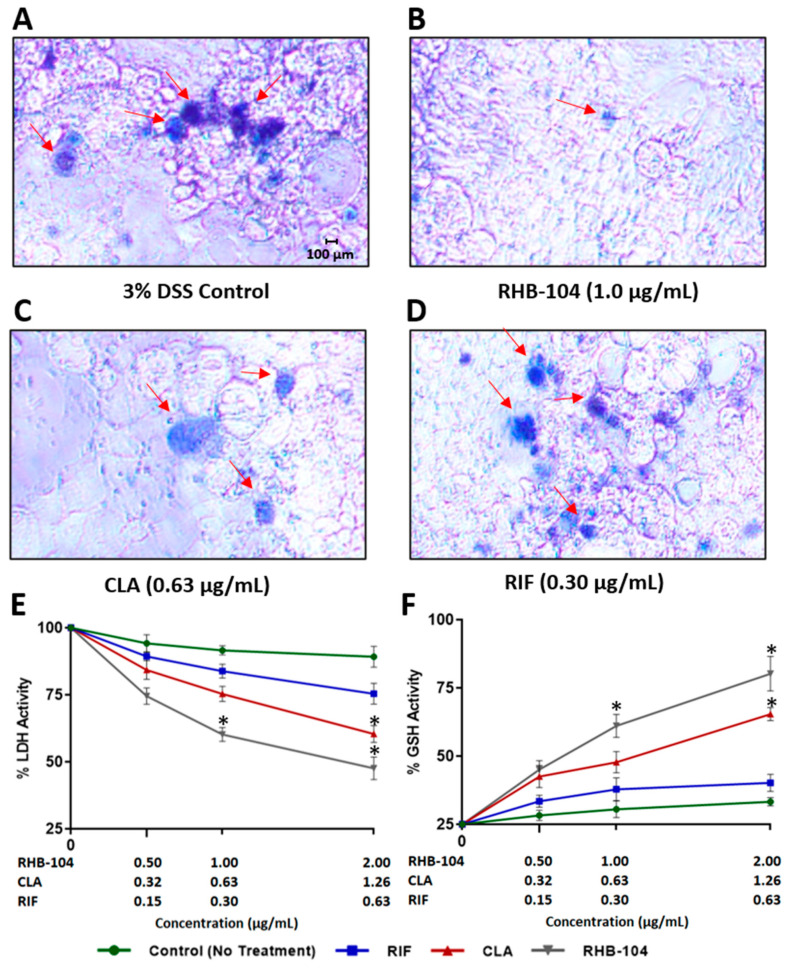
Anti-cytotoxic activity of RHB-104 and its major components in Caco-2 monolayer treated with 3% DSS. Trypan blue exclusion assay showing the cytotoxic effects (indicated by red arrows) in DSS-treated Caco-2 monolayers (**A**), and DSS-treated Caco-2 monolayers with 1.0 μg/mL RHB-104 (**B**), 0.63 μg/mL CLA (**C**), and 0.30 μg/mL RIF (**D**). LDH Activity (**E**) and GSH activity (**F**) in DSS-treated Caco-2 monolayers treated with RHB-104 (0, 0.5, 1.0, 2.0 μg/mL), CLA (0, 0.32, 0.63, and 1.26 μg/mL), and RIF (0, 0.15, 0.30, 0.60 μg/mL) for 24 h. * *p* < 0.05.

**Figure 8:**
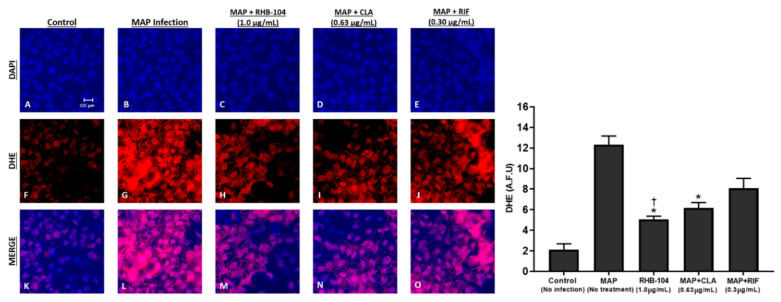
Oxidative stress levels demonstrated by DHE fluorescence staining assay following treatment with RHB-104 and its major components in MAP infected Caco-2 Monolayers. Total nuclei are stained with DAPI in blue (**A**–**E**). DHE positive cells are stained in red (**F**–**J**), and merged cells are presented in pink (**K**–**O**). The histogram shows quantitative corrected DHE fluorescence integrated density in control and treated groups. * *p* value < 0.05 compared to no treatment control. † *p* value < 0.05 compared to no treatment control and MAP + RIF values.

The viability of MAP-infected or DSS-treated Caco-2 monolayers was detected with a trypan blue exclusion assay. The test demonstrated a clear reduction in cell death upon exposure of monolayers to RHB-104 (1.0 μg/mL), followed by CLA (0.63 μg/mL), and then RIF (0.30 μg/mL), which only had a minimal effect on preventing cell death (Figure 6A–D and Figure 7A–D). To further confirm our findings, we assessed the effect of RHB-104 (0.5, 1.0, and 2.0 μg/mL) and its individual components, CLA (0.32, 0.63, and 1.26 μg/mL) and RIF (0.15, 0.30 and 0.60 μg/mL), on tissue damage and oxidative stress activity markers, LDH and GSH, in Caco-2 monolayers following 24 h of MAP infection or 3% DSS treatment. The groups infected with MAP or treated with DSS without any antibiotics showed the highest levels of LDH and the lowest levels of GSH, and were therefore used as reference controls. RHB-104 significantly decreased LDH levels at 1.0 and 2.0 μg/mL (50.32 ± 3.9% and 41.68 ± 2.4%, respectively) in MAP-infected monolayers (Figure 6E). In addition, Figure 7E showed that RHB-104 at 1.0 and 2.0 μg/mL had similar effects on LDH levels in DSS-treated monolayers (60.23 ± 2.6% and 47.61 ± 4.2%, respectively), whereas CLA only significantly decreased LDH activity levels at 1.26 μg/mL (52.47 ± 5.3% and 60.41 ± 3.1, respectively) in both MAP-infected and DSS-treated monolayers (Figure 6E and Figure 7E). Similarly, the antioxidant effect of RHB-104 was significantly higher than its individual components by increasing GSH levels at 1.0 and 2.0 μg/mL (75.41 ± 3.3% and 87.27 ± 2.3%, respectively) in MAP-infected monolayers, and similar effects were found at 1.0 and 2.0 μg/mL (61.09 ± 4.2% and 80.23 ± 6.3%, respectively) in DSS-treated monolayers (Figure 6F and Figure 7F). The effect of CLA on GSH activity was only significant at 1.26 μg/mL in both MAP-infected and DSS-treated monolayers (70.19 ± 6.7% and 65.43 ± 2.4, respectively) (Figure 6F and Figure 7F). In contrast, RIF did not show any significant effects on LDH or GSH activity in either MAP-infected or DSS-treated monolayers at all three tested concentrations (Figure 6E,F and Figure 7E,F). Oxidative stress level in MAP-infected fully differentiated Caco-2 monolayers was also measured by DHE fluorescence staining assay (Figure 8). MAP infection caused the highest level of oxidative stress, whereas RHB-104 (1.0 μg/mL) and CLA (0.63 μg/mL) caused significant reductions in oxidative stress levels compared to MAP control. Additionally, RHB-104 caused a significantly greater reduction in oxidative stress levels compared to RIF (0.30 μg/mL) and a slight decrease in oxidative stress levels compared to CLA (0.63 μg/mL), while CLA did not cause a significant reduction in oxidative stress levels compared to RIF (0.30 μg/mL).

### 4.7. RHB-104 Most Effectively Reduces T-cell Proliferation in Phytohemagglutinin (PHA) and MAP PPD-Treated Cells

We measured T-cell proliferation in PHA- and MAP PPD-treated T-cells in the presence and absence of treatment with 1.0 μg/mL RHB-104, 0.63 μg/mL CLA, and 0.30 μg/mL RIF (Table 4). T-cell proliferation was induced by PHA or MAP PPD, which caused relative T-cell proliferation levels of 3.60 ± 0.059 and 3.12 ± 0.087, respectively, compared to cells without these treatments. Relative to the PHA control, it was found that RHB-104, CLA, and RIF significantly lowered T-cell proliferation in PHA-treated cells (0.75 ± 0.002, 1.09 ± 0.021, and 0.99 ± 0.013, respectively). Likewise, RHB-104, CLA, and RIF also significantly lowered T-cell proliferation in MAP PPD-treated cells relative to the MAP PPD control (1.09 ± 0.010, 1.76 ± 0.060, and 1.86 ± 0.034, respectively). Furthermore, the reductions in T-cell proliferation caused by RHB-104 in both PHA- and MAP PPD-treated cell were significantly higher than that caused by CLA or RIF treatment.

## 5. Discussion

Our study is the first to report the synergistic immunomodulatory and anti-inflammatory effects of RHB-104 independent of its bactericidal activity, thus further supporting its use as a treatment for CD patients. There is an urgent need for improved treatments for CD, which is characterized by high degrees of inflammation and a possible association with MAP. RHB-104 drug formulation serves as a promising therapeutic strategy to alleviate disease symptoms in these patients, even in the absence of MAP detection.

Abnormal accumulation of macrophages, granulocytes, lymphocytes, and platelets is a well-known characteristic of many inflammatory conditions regardless of their infective or non-infective origins. Consequently, this leads to higher levels of pro-inflammatory cytokine release, oxidative stress, and local tissue damage. There has been greater emphasis on investigating the potential anti-inflammatory role of antibiotics in reducing symptoms of these conditions [1]. A potentially more effective approach to treat inflammatory conditions associated with bacterial infections is to use a therapeutic compound that has both antimicrobial and anti-inflammatory mechanisms.

Macrolides are a class of antibiotics that have a broad spectrum of activity against gram-positive and some gram-negative bacteria. Specifically, CLA is active against nontuberculous mycobacteria, including MAP and *M. smegmatis* [23]. Additionally, CLA attenuates the production of pro-inflammatory cytokines such as IL-2 and IL-8, which is well-documented both in in vitro and in vivo studies [24,25]. Studies have confirmed that CLA exhibits potent anti-angiogenic activity through inhibition of vascular endothelial growth factor (VEGF) [26,27,28]. Macrolides also have unique and favorable pharmacokinetic properties [29]. For instance, the concentration of CLA in epithelial lung tissue after administration of a certain oral dose is 10-fold higher than the plasma concentration level [29]. Moreover, the concentration of macrolides in inflammatory cells tends to be more than 100-fold higher than that in extracellular fluid, which enables phagocytic cells to deliver cumulative levels of drugs to sites of infection [30].

The use of rifamycins in the treatment of rheumatoid arthritis (RA) is an interesting application that also showed an additional anti-inflammatory activity to be associated with this class of antibiotics [31]. To explain this anti-inflammatory mechanism, a study showed that a reduction in neutrophil locomotion is caused by rifamycin SV in a concentration-dependent manner [32]. In addition, rifaximin has been used to suppress the inflammatory response in gastrointestinal disorders such as inflammatory bowel syndrome (IBS) [33]. Direct intracolonic administration of rifamycin SV reduced inflammation in dinitrobenzene sulfonic acid-induced colitis in an animal model [34]. Therefore, combining a macrolide, such as CLA, with a rifamycin antibiotic, such as RIF, is expected to have promising synergistic immunomodulatory effects. This could be a useful treatment for several inflammatory conditions, especially those associated with microbial infection, such as CD.

We have previously evaluated the proprietary RHB-104 capsule formulation against a broad spectrum of microorganisms associated with CD including MAP, *Listeria monocytogenes*, and *Staphylococcus aureus* [23]. We determined the synergistic effect of combining CLA, RIF, and CLO in a single capsule triple formulation, which has a lower MIC compared to its individual components [23]. This intrigued us to evaluate the anti-inflammatory and immunomodulatory effects of RHB-104 and its two major ingredients, CLA and RIF, in vitro to find out if there is a synergistic anti-inflammatory effect induced by combining these three antibiotics. We did not test the effect of CLO individually because it makes up only 6.7% of the RHB-104 formulation.

**Figure 9:**
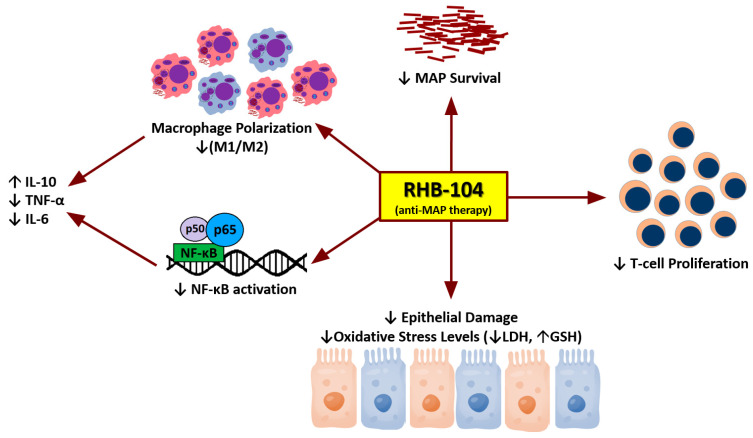
Schematic diagram summarizing the immunomodulatory activity of RHB-104.

This study confirmed the bactericidal effects of RHB-104 in MAP-infected macrophages and investigated the immunomodulatory and anti-inflammatory effects of RHB-104 and its major components. This was achieved by comparing the effects of RHB-104, CLA, and RIF on M1/M2 macrophage polarization, NF-κB activation, inflammatory gene expression and protein levels, anti-cytotoxicity, and T-cell proliferation, all of which have been reported to be increased in CD patients [19,20,21,22]. Since MAP may not be present in all CD cases, LPS was used to induce an inflammatory response that can be likened to that seen in CD patients as it causes an increase in NF-κB activation and pro-inflammatory cytokine expression. This also allowed us to identify the anti-inflammatory properties of RHB-104 independent of its bactericidal activity (Figure 9).

Our results showed that RHB-104 exhibits synergistic anti-inflammatory effects since it caused the greatest reduction in the M1/M2 ratio in a dose-dependent manner compared to its major components. NF-κB p65 activation was also most strongly reduced in response to RHB-104 in MAP-infected and LPS-treated macrophages as compared to its major components, CLA and RIF. NF-κB is responsible for regulating transcription of the pro-inflammatory cytokines, TNF-α and IL-6, which are upregulated in CD patients [19,20]. In agreement with this, our results showed that both TNF-α and IL-6 gene and protein expression were most significantly reduced by RHB-104 in a dose-dependent manner as compared to its major components in both MAP-infected and LPS-treated macrophages. The synergistic anti-inflammatory effect caused by RHB-104 may be due to the combination of CLA and RIF within the triple antibiotic formulation which have been shown to independently inhibit NF-κB activation [4,9]. Gene and protein expression of the anti-inflammatory cytokine, IL-10, was also significantly upregulated only in response to RHB-104, but not by treatment with CLA or RIF.

The presence of MAP infection causes systemic inflammation and oxidative stress, which is characterized by higher levels of glutathione peroxidase (GPx) enzymatic activity [35]. Therefore, we assessed tissue damage induced by either DSS or MAP infection in intestinal tissue culture, which was most significantly reduced by the presence of RHB-104 treatment as compared to its major components. We used both MAP and DSS to confirm that the reduction in tissue damage caused by RHB-104 was not dependent on anti-MAP activity. Using Caco-2 monolayers, which serve as an effective in vitro model for the intestinal barrier in CD patients, our results showed reduced LDH activity and increased GSH activity, where LDH is a marker of tissue damage, and GSH is an antioxidant that is correlated with reduced oxidative stress. As concluded previously, the synergistic effect of RHB-104 was higher than the effects seen by its major components. In the aforementioned results, the effect of RHB-104 was stronger in MAP-infected cells compared to LPS- or DSS-treated cells and this is likely because the bactericidal effects of RHB-104 against MAP contributed to the reduction in inflammation observed. However, the anti-inflammatory effects of RHB-104 were confirmed to be independent of its bactericidal activity since inflammation was reduced even in the absence of MAP. The production of reactive oxygen species (ROS), as indicated by DHE staining, was also reduced in MAP-infected Caco-2 monolayers treated with RHB-104. Finally, our results also showed that RHB-104 caused the greatest reduction in T-cell proliferation compared to both CLA and RIF following PHA and MAP PPD stimulation. As CD is characterized by elevated levels of T-cells in the mucosa, the modulation of T-cell apoptosis is expected to have therapeutic effects [36].

The current recommended CD treatment guidelines are based primarily on traditional immunosuppressive therapeutics such as azathioprine and biological drugs targeting specific pro-inflammatory cytokines [37]. Nevertheless, up to 40% of CD patients are categorized as non-responsive to primary treatment, which suggested that these patients should be investigated for MAP infection and treated with antibiotics accordingly [38,39,40]. Currently, RHB-104 triple antibiotic combination is the only medicinal product directed against MAP infection, and it is still under clinical development [8]. The ongoing MAP US global multicenter phase III clinical trial is using a fixed oral daily dose of RHB-104 to determine its safety and efficacy in treating moderate to severe active CD over 26 to 52 weeks, considering disease remission as a primary endpoint [41]. Recent clinical trial reports have demonstrated that RHB-104 has shown a consequential improvement in CD biomarkers of inflammation such as C-reactive protein (CRP) and Crohn’s disease activity index (CDAI) with or without concomitant anti-TNF-α or immunosuppressive therapy [17].

Achieving full eradication of mycobacterial infection is considered a relatively challenging task, which is due to the presence of cell wall deficient forms, slow growth rate, and their capability of intracellular survival through inhibition of macrophage apoptosis [42,43]. Moreover, MAP can evade host immune detection through several steps of evolved mechanisms, which complicates its baseline diagnostic results before initiation of antibiotics treatment [44]. However, the presence of mycobacteria in clinical samples does not necessarily mean that it should be accompanied with clinical pathophysiology, as only 4% of latent tuberculosis cases worldwide develop disease-related symptoms [45]. Therefore, the presence of MAP infection in CD patients who went through antibiotics treatment could explain why these patients continued testing positive for MAP despite the fact that their CD symptoms were alleviated. In this context, our data indicates that RHB-104’s immunomodulatory therapeutic properties could be useful for treating CD patients regardless of MAP infection status.

## Figures and Tables

**Table 1:** RT-PCR primer sequences for tested genes.

Gene	Forward Primer Sequence (5′→3′)	Reverse Primer Sequence (5′→3′)
*GAPDH*	5′-CTTTTGCAGACCACAGTCCATG-3′ (22 bases)	5′-TTTTCTAGACGGCAGGTCAGG-3′ (21 bases)
*iNOS*	5′-GGAGCAACGTTGAGGAAATAAGACT-3′ (25 bases)	5′-AAGAGCCAGAAGCGCTATCAC-3′ (21 bases)
*CD206*	5′-GGAGGATTCCATGTATTTGTGAGC-3′ (24 bases)	5′-AAATGAGTGAAGTGAAATCAGTTACCT-3′ (27 bases)
*TNF-α*	5′-TCTCCCTCAAGGACTCAGCTTTCTG-3′ (25 bases)	5′-TGAGAGGAAGAGAACCTGCCTGG-3′ (23 bases)
*IL-6*	5′-CAGGAGAAGATTCCAAAGATGTAGCC-3′ (26 bases)	5′-TGCTCTAGAACCCAGCAAAGAC-3′ (22 bases)
*IL-10*	5′-ATGTCTAGTTCAGGCAGTCCCA-3′ (22 bases)	5′-GGGCTTGCTCTTGCAAAACC-3′ (20 bases)

**Table 2:** NF-κB p65 activation scores in macrophages infected with MAP or treated with LPS following treatment with RHB-104 and its major components.

Treatment	Antibiotic Concentration (µg/mL)	NF-κB p65 Activation Score (%)
Positive Control	-	100.0
Negative Control	-	6.9
MAP + RHB-104	0.50	48.4
	1.00	21.5
	2.00	13.4
MAP + CLA	0.32	58.3
	0.63	49.6
	1.26	24.1
MAP + RIF	0.15	83.8
	0.30	73.1
	0.60	57.5
LPS + RHB-104	0.5	67.2
	1.0	37.8
	2.0	23.3
LPS + CLA	0.32	68.2
	0.63	57.2
	1.26	33.6
LPS + RIF	0.15	89.1
	0.30	83.4
	0.60	68.7

**Table 3:** Measurement of *TNF-α*, *IL-6*, and *IL-10* cytokines in macrophages infected with MAP or treated with LPS following treatment with RHB-104 and its major components.

Treatment	Antibiotic Concentration (µg/mL)	TNF-α ± SD (pg/mL)	IL-6 ± SD (pg/mL)	IL-10 ± SD (pg/mL)
MAP Control	-	191.2 ± 8.6	201.8 ± 11.7	61.9 ± 9.8
LPS Control	-	217.4 ± 5.1	224.3 ± 9.6	52.2 ± 7.4
No Treatment	-	75.7 ± 6.5	82.9 ± 5.1	100.4 ± 6.5
MAP + RHB-104	0.50	175.3 ± 8.4	182.2 ± 8.1	79.2 ± 2.9
	1.00	123.7 ± 6.7 *	139.4 ± 3.2 *	88.6 ± 3.5 *
	2.00	95.8 ± 1.8 *	117.5 ± 2.5 *	95.1 ± 5.8 *
MAP + CLA	0.32	184.7 ± 3.6	191.5 ± 8.3	68.3 ± 3.1
	0.63	165.8 ± 5.1	173.6 ± 3.1	75.4 ± 2.7
	1.26	146.1 ± 6.2 *	153.2 ± 4.6 *	80.3 ± 8.7
MAP + RIF	0.15	187.2 ± 5.3	197.3 ± 7.2	63.6 ± 4.1
	0.30	179.1 ± 1.9	184.9 ± 5.9	70.2 ± 2.1
	0.60	166.4 ± 8.3	172.8 ± 3.9	77.3 ± 3.4
LPS + RHB-104	0.5	179.3 ± 2.0	182.6 ± 5.1	76.4 ± 6.3
	1.0	139.7 ± 4.7 *	151.1 ± 2.5 *	84.3 ± 6.9 *
	2.0	110.9 ± 4.0 *	137.2 ± 2.7 *	91.1 ± 5.2 *
LPS + CLA	0.32	185.8 ± 1.6	193.3 ± 4.4	61.2 ± 4.2
	0.63	162.4 ± 6.2	174.8 ± 3.6	69.7 ± 7.8
	1.26	158.2 ± 7.3 *	169.7 ± 1.1 *	71.9 ± 6.9
LPS + RIF	0.15	188.3 ± 3.1	206.8 ± 8.0	54.6 ± 2.3
	0.30	179.7 ± 8.9	192.4 ± 1.3	62.4 ± 7.2
	0.60	166.1 ± 5.6	182.1 ± 1.6	67.5 ± 4.9

* *p* < 0.05.

**Table 4:** T-cell proliferation following treatment with MAP purified protein derivative (PPD) or phytohemagglutinin (PHA) and RHB-104, and its major components.

Treatment	T-cell Proliferation (OD at 450 nm)	Relative T-cell Proliferation
Control (no PHA or MAP PPD)	0.061	1.00 ± 0.003
PHA Control	0.219	3.60 ± 0.059
PHA + 1.0 µg/mL RHB-104	0.046	0.75 ± 0.002 *^,†^
PHA + 0.63 µg/mL CLA	0.066	1.09 ± 0.021 *
PHA + 0.30 µg/mL RIF	0.060	0.99 ± 0.013 *
MAP PPD Control	0.189	3.12 ± 0.087
MAP PPD + 1.0 µg/mL RHB-104	0.066	1.09 ± 0.010 *^,†^
MAP PPD + 0.63 µg/mL CLA	0.107	1.76 ± 0.060 *
MAP PPD + 0.30 µg/mL RIF	0.113	1.86 ± 0.034 *

* *p* value < 0.05 compared to PHA or MAP PPD control; ^†^ *p* value < 0.05 compared to CLA and RIF treatment.
